# Current Approaches and Future Perspectives for Nanobodies in Stroke Diagnostic and Therapy

**DOI:** 10.3390/antib8010005

**Published:** 2019-01-03

**Authors:** Larissa Jank, Carolina Pinto-Espinoza, Yinghui Duan, Friedrich Koch-Nolte, Tim Magnus, Björn Rissiek

**Affiliations:** 1 Department of Neurology, University Medical Center Hamburg-Eppendorf, 20246 Hamburg, Germany; larissajank@gmail.com (L.J.); yinghui.duan@stud.uke.uni-hamburg.de (Y.D.); t.magnus@uke.de (T.M.); 2 Institute of Immunology, University Medical Center Hamburg-Eppendorf, 20246 Hamburg, Germany; c.pinto@uke.de (C.P.-E.); nolte@uke.de (F.K.-N.)

**Keywords:** nanobodies, ischemia, stroke, MCAO, single domain antibodies

## Abstract

Antibody-based biologics are the corner stone of modern immunomodulatory therapy. Though highly effective in dampening systemic inflammatory processes, their large size and Fc-fragment mediated effects hamper crossing of the blood brain barrier (BBB). Nanobodies (Nbs) are single domain antibodies derived from llama or shark heavy-chain antibodies and represent a new generation of biologics. Due to their small size, they display excellent tissue penetration capacities and can be easily modified to adjust their vivo half-life for short-term diagnostic or long-term therapeutic purposes or to facilitate crossing of the BBB. Furthermore, owing to their characteristic binding mode, they are capable of antagonizing receptors involved in immune signaling and of neutralizing proinflammatory mediators, such as cytokines. These qualities combined make Nbs well-suited for down-modulating neuroinflammatory processes that occur in the context of brain ischemia. In this review, we summarize recent findings on Nbs in preclinical stroke models and how they can be used as diagnostic and therapeutic reagents. We further provide a perspective on the design of innovative Nb-based treatment protocols to complement and improve stroke therapy.

## 1. Stroke and Post-Stroke Inflammation

According to the WHO Global Health, strokes are the second leading cause of death worldwide (10.2% of all deaths in 2016) and the second leading cause for loss of healthy years (5.2% of all disability-adjusted life years in 2016). In the future, these numbers are expected to further increase. In upper-middle income countries, prevalence is increasing due to the aging population [1], while in low-income countries, stroke incidence is rising due to changes in lifestyle and lack of adequate risk factor management [2]. Ischemic stroke is characterized by a reduced blood supply to the brain parenchyma. The following four underlying causes each account for about 25% of the ischemic strokes: (1) Embolization of a cardiac thrombus, (2) occlusion of a large vessel with atherosclerotic lesions, (3) small vasculature pathology usually leading to lacunar infarcts, and (4) other causes [3,4]. Due to the reduced blood flow, there is an energy deficit in neuron as well as a build-up of cellular waste products, such as lactate. This causes ionic disbalance, inducing the release of neurotransmitters, notably glutamate [5]. Glutamate binds to ionotropic glutamate receptors on neurons and calcium accumulates intracellularly. The calcium overload activates enzymatic cascades involved in neuron necrosis and apoptosis. These enzymes include phospholipases compromising membrane integrity as well as proteases mediating cell death and mitochondrial reactive oxygen species (ROS) production [6,7]. Furthermore, lack of adenosine triphosphate (ATP) reduces the activity of Na^+^/K^+^ ATPase, inducing neuronal edema [8].

Ischemic damage to neurons and tissue necrosis in the infarct core involves the release of damage associated molecular patterns (DAMPs) into the extracellular space, such as high mobility group protein B1 [9], ATP [10], heat shock protein 70 [11,12], histones, and DNA [13]. Extracellular DAMPs can bind to pattern recognition receptors (including the receptor for advanced glycation end products (RAGE), P2X7, and Toll-like receptors) on brain resident innate immune cells such as microglia, initiating an innate immune response within the first minutes after vessel occlusion [14]. In the first hours following stroke onset, microglia activation orchestrates the infiltration of other mononuclear cells in the peri-infarcted region, the penumbra [15]. The main functions of microglia include initiation and amplification of sterile inflammation by releasing proinflammatory cytokines (tumor necrosis factor α (TNFα), IL-1β and IL-6), generating ROS and nitric oxide (NO), phagocytosis to clear cell debris, and attracting peripheral immune cells to the penumbra with cytokines and chemokines, including monocyte chemoattractant protein 1 (MCP-1), macrophages inflammatory protein 1 α (MIP-1α), and CXCL-8 [16,17,18,19]. Three days post-ischemia, the influx of peripheral immune cells is at its maximum [15]. Neutrophils are the most abundant peripheral immune cell population in the ischemic brain, which further enhance the sterile inflammation and contribute to infarct size growth [14]. At the peak of peripheral immune cell infiltration, T-cells are also attracted to the penumbra. CD4^+^ and CD8^+^ T-cells are involved in a major histocompatibility complex (MHC) dependent, i.e., antigen specific adaptive immune response, while more innate-like lymphocyte populations, such as γδ T-cells, NKT cells, and NK cells are activated by cytokines and other molecules of the inflammatory milieu. This heterogeneous population of cells can contribute to infarct size growth either directly by cell-cell interactions, or indirectly through the induction of a humoral immune response or the release of cytotoxic substances [14,20].

To reach the penumbra, the attracted leukocytes need to cross the blood brain barrier (BBB). This structure consists of a monolayer of brain endothelial cells (ECs) surrounded by a basal membrane, pericytes, and astrocytes [21]. Proinflammatory cytokines released during cerebral ischemia activate ECs, leading to an increase in vesicles for transcellular transport and an increase in cell surface molecules associated with leukocyte recruitment [22] e.g., P-selectin and intercellular adhesion molecule 1 (ICAM-1), which mediate leukocyte rolling and adhesion, respectively [23,24]. Furthermore, matrix metalloproteases (MMPs) released in the penumbra change the tight junction conformation, enabling paracellular transport across the BBB [25].

## 2. Nanobodies—Single Domain Antibodies

### 2.1. Structure of Nanobodies and Conventional Antibody Fragments

**Figure 1:**
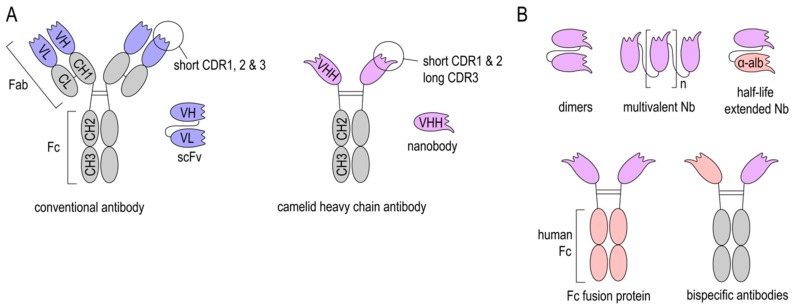
Structure of Nanobodies. (**A**) Structure of conventional antibodies (cAb), single-chain variable fragments (scFv), heavy-chain antibody (HcAb), and Nanobodies (Nbs). (**B**) Nbs can be produced as dimers and multimers to improve binding to their target or linked to an anti-albumin Nb to increase their in vivo half-life. Latter can be also achieved by fusing an Fc region of e.g., human IgG. Further, bispecific Nb-Fc-fusion proteins can also be expressed.

Camelids, nurse sharks, and spotted ratfish exhibit naturally occurring heavy-chain-only antibodies (HcAbs) (Figure 1A). Interestingly, in camelids, HcAbs have evolved from conventional antibodies (cAbs), suggesting that they exhibit certain functional characteristics that are missing in cAbs [26]. This might be attributed to their smaller size and unique structure. Immunoglobulin G (IgG), the most abundant serum antibodies isotype in humans, consists of two heavy chains with three constant (CH1-3) and one variable domains (VH) each and two light chains with one constant (CL) and one variable domain (VL) each. In contrast, HcAbs only contain two heavy chains with two constant and one variable heavy-chain domain (VHH). Therefore, antigen binding by HcAbs is reduced to the VHH domain [27,28].

Both cAbs and HcAbs can be fragmented into smaller antigen-binding subunits in order to improve their tissue penetration. Common IgG modifications include: Cleavage of the Fc region to obtain Fab fragments, fusion of VH and VL domains with linker peptides to obtain single chain variable fragments (scFv), and the generation of autonomous human heavy-chain variable fragments (VH) [29,30].

VHH domains derived from HcAbs can be expressed as recombinant proteins, termed “Nanobodies” (Nbs), since they are one-tenth of the molecular size of an IgG molecule (Nbs: 15 kDa and IgG: 150 kDa). Nbs consists of four conserved framework regions and three antigen-binding loops, known as the complementarity determining regions (CDRs). The particularly long CDR3 rends the paratope its convex shape, building protrusions that can reach cryptic epitopes often not accessible to cAbs [27,28,31,32].

The first step in Nb generation is usually the immunization of HcAb-bearing large animals, such as llamas, alpacas, or sharks, followed by multiple boost immunizations in order to achieve an enrichment of high-affinity binders [33,34]. To overcome some of the logistical and financial limitations associated with immunization of large camelids, mice producing heavy-chain antibodies are being generated [35].

### 2.2. Advantages and Limitations of Nanobodies

One major advantage of Nbs is their small molecular size, which enables good tissue penetration and distribution. Furthermore, Nbs can refold after certain denaturation processes. This makes them very stable at extreme temperatures, low pH, and in the presence of proteases [36,37,38]. Additionally, Nbs are highly soluble in aqueous solutions, even at high concentrations [39,40]. These properties facilitate different routes of administration (e.g., intravenous, intraperitoneal, intrathecal, etc.), as well as various sites of action, such as pathological micro milieus. Due to their relatively simple structure, Nbs can be optimized by genetic engineering to obtain desired properties. They can be genetically linked to anti-albumin Nbs (Figure 1B) to extend their in vivo half-life. Further, Nb-Fc-fusion proteins allow binding to Fc receptors [41]. Several of these genetic modifications are aiming at facilitating crossing of the blood-brain-barrier (BBB) and are discussed below.

The small size of Nbs allows good tissue penetration, and also accounts for their short in vivo half-life when injected into experimental animals or into humans, since monovalent Nbs (≈15 kDa) are rapidly eliminated via the kidney (70 kDa cut-off for renal filtration in humans) [42]. Though this might be beneficial for short-term applications, such as molecular imaging, it also is considered to be detrimental in long-term therapeutic applications. Increasing the size of Nbs through dimerization/multimerization, fusion to an anti-albumin Nb [43,44] or Fc engineering [45,46] can increase their serum half-life (Figure 1B). However, an increase in size and change in structure may also affect tissue penetration, affinity, stability, and solubility of Nbs. Another limitation of Nbs is that they are potentially immunogenic in humans since they originate from camelid species. Though recombinant Nbs lack an Fc region and share a large sequence identity with the human VH of family 3 [47], the risk of eliciting an anti-Nb adoptive immune response increases upon repeated application. Humanization of Nbs is a strategy to address this problem, but it does not always sufficiently prevent antidrug antibody responses. Repeated injections of humanized Nbs (Caplacizumab) against von Willebrand Factor (vWF) resulted in a low incidence (9%) of antidrug antibody responses in the TITAN phase II study [48,49], while a clinical trial with a humanized anti-DR5 Nb (TAS266) had to be terminated because the applied Nbs evoked adverse host immune responses [50]. To this end, human scFvs or mutated human IgG lacking Fc-mediated effector functions have to be considered as a nonimmunogenic alternative to Nbs. However, for stroke diagnostic and therapy e.g., modulation of post-stroke inflammation, a single application of Nbs early after stroke onset might not reach the threshold for inducing an anti-Nb adaptive immune response. Since this threshold is highly dependent on the individual Nb, future studies on the application of Nbs in stroke should also address the issue of immunogenicity.

### 2.3. Nanobodies at the BBB

**Figure 2:**
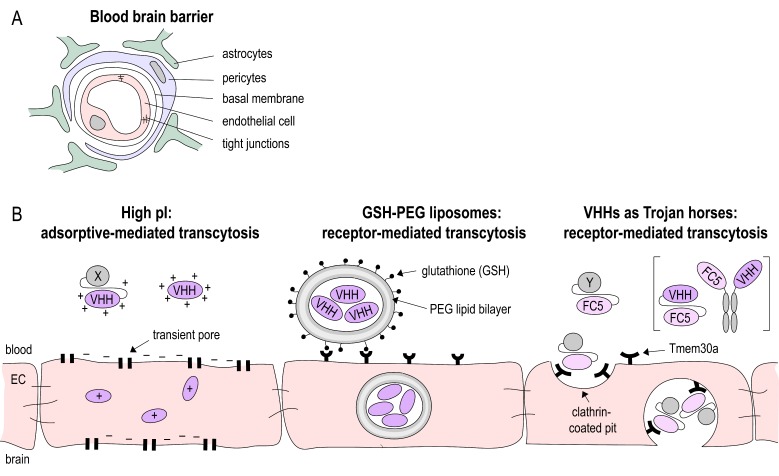
Blood-brain-barrier (BBB) crossing Nanobodies. (**A**) The BBB is built by the neurovascular unit consisting of endothelial cells connected via tight junctions, a basal membrane, pericytes and astrocytes foot processes. (**B**) Various strategies have been applied to shuttle Nbs (VHH) across the BBB: increasing the isoelectric point (pI) to facilitate uptake by endothelial cells (EC); package of Nbs in glutathione coated liposomes and receptor-mediated uptake into EC. Nbs such as the Tmem30a-specific Nb FC5 that target EC membrane receptors can act as Trojan horse to shuttle other Nbs or peptides across the BBB.

A major challenge for brain-targeting biologics is crossing of the BBB (Figure 2A). Under physiological conditions, only a very small fraction of intravenously injected cAbs cross the BBB (IgG CNS/plasma ratio: 0.1–1%), and once reaching the brain parenchyma, they are rapidly cleared by FcRn mediated efflux [51,52]. Nbs, on the other hand, lack an Fc region, are smaller in size, and more stable, promising facilitated delivery to the brain. However, when administered under non-pathological conditions, monovalent Nbs do not reach sufficient concentrations for in vivo brain imaging [53] and therapeutic purposes [42]. Pierre Lafaye’s group was able to show that the brain penetration of Nbs can be improved by exploiting the process of adsorptive mediated transcytosis [54,55]. This transcytosis mechanism has been earlier identified to shuttle basic proteins and peptides across the BBB [56]. The basicity of a Nb can be increased by exchanging the carboxyl groups of the Nb with positively charged amino groups, thereby increasing the isoelectric point. The same group developed Nb-fluorochrome constructs (with pI = 8.3 and 9.5) that successfully label targets in an Alzheimer’s disease model after being administered intravenously [57]. Nevertheless, high Nb concentrations (10–50 mg/kg) are required for detection and Nbs were only detectable for 4 h post-injection, suggesting a half-life too short for therapeutic purposes. The latter issue can be addressed by extending the half-life of Nbs. However, there is some controversy about the benefit of half-life extended Nbs for brain targeting. Iqbal et al. showed that fusion of an anti-EGFR Nb to a human Fc fragment improved the imaging of brain tumors [58], while another study of the kinetics of Nb-Fc fusion proteins showed that despite the extended serum half-life, the modification did not improve delivery across the BBB [59].

An alternative approach to deliver drugs to the brain is by receptor mediated transport (Figure 2B). Therapeutics are linked to ligands of or antibodies against receptors that are highly expressed on the BBB, such as the transferrin receptor [60,61], the insulin receptor [62,63], or the low-density lipoprotein receptor-related protein [64]. This antibody-mediated delivery of therapeutic proteins or peptides was studied in various neurological diseases, including stroke [65,66]. However, to date, there is only a limited number of studies in which therapeutic Nbs are delivered across the BBB via receptor mediated transcytosis. Rotman et al. loaded anti-amyloid Nbs into glutathione PEGylated liposomes. Glutathione can bind to receptors on cerebral endothelial cells and by this the liposomes are transported across the BBB [59].

Furthermore, Nbs that facilitate receptor mediated transport of biologics have been generated. The Nb clone FC5 was generated by phage-display in order to select Nbs that transmigrate across human cerebromicrovascular endothelial cells [67]. Later, it was discovered that FC5 targets a luminal sialoglycoprotein receptor (TMEM-30A), which induces the formation of clathrin vesicles and ultimately transcytosis [68]. By this mechanism, FC5 can act as a Trojan horse, transporting attached molecules across the BBB. Webster et al., for instance, generated FC5-Fc fusion proteins and conjugated these with the analgesic peptides dalargin and neuropeptide Y to deliver them across the BBB. Brain penetration of the FC5-Fc proteins was up to 30-fold higher compared to Fc protein alone [46]. The same group created bispecific antibodies with one FC5-arm and one arm targeting the metabotropic glutamate receptor-1 (mGluR1). These bispecific constructs showed a 20-fold higher brain penetration than unmodified anti-mGluR1 IgG [69].

## 3. Stroke Imaging—New Job Opportunities for Nanobodies?

### 3.1. Principles of Stroke Imaging

In stroke therapy, early intervention by thrombolysis or mechanical thrombectomy is essential to save hypoxic tissue. However, the mere assessment of clinical signs and symptoms of stroke are not sufficient for diagnosis. Hence, imaging plays an important role in stroke diagnostics and management.

Acute imaging has to be fast and rule out other possible diagnoses, such as intracerebral bleeding, or so-called stroke mimics (e.g., epileptic seizures or migraine) [70]. The current standard procedure is computed tomography (CT) or magnetic resonance imaging (MRI) (if applicable with angiograms) within the first 4.5 h of stroke onset [71]. In the subacute stage, imaging reveals risk factors of cerebrovascular events, such as atherosclerotic plaques or dissections, in order to initiate adequate secondary prevention. To this end, imaging of the extracranial and intracranial arteries, the aorta and the heart is performed with CTA, MRA, carotid Doppler ultrasonography and echocardiography.

Anatomical imaging modalities, such as conventional CT and MRI, detect the secondary consequences of post-ischemic inflammation e.g., changes in diffusion and edema. Molecular and cellular imaging techniques, on the other hand, can be used to visualize and quantify distinct molecules, cell populations and processes. Here, we will focus on antibody- and Nb-assisted molecular imaging.

### 3.2. Imaging Endothelial Activation

Most studies on antibody-mediated molecular imaging in stroke target endothelial markers. These molecules are upregulated directly after occlusion and the antibody can bind these epitopes without crossing the BBB [72]. For example, Quenault et al. used microparticle of iron oxide (MPIOs) coated with P-selectin-targeting antibodies to identify transient ischemic attacks and to exclude other differential diagnosis, such as epilepsy and migraine, in MRI scans [73]. Other known endothelial activation markers used for antibody-mediated MRI stroke imaging include vascular cellular adhesion molecule 1 (VCAM-1) [74,75], platelet and endothelial cell adhesion molecule 1 (PECAM-1) [76], and ICAM-1 [77].

MRI is the modality of choice because it combines desirable properties, including relatively fast acquisition times, easy accessibility, and high safety. Nevertheless, molecular, nuclear, and optical imaging are important alternatives due to their high sensitivity. However, each imaging modality has its own drawbacks, including radiation for nuclear and CT imaging, possible tissue accumulation of MRI contrast agents, and limited imaging depth for optical imaging. Besides imaging-based limitations, cAb-mediated imaging may cause further difficulties in clinical application, including their long serum half-life (1–3 weeks), and therefore, strong background signal [78]. This could be addressed by replacing cAbs with Nbs. Devoogdt’s group, for instance, created Nb-based imaging probes for positron-emission tomography (PET)/CT [79] and single photon emission computed tomography (SPECT) [80] targeting VCAM-1 for atherosclerosis plaque risk assessment. It is worth noting that Nbs unite several beneficial characteristics for endovascular imaging, including a high affinity to withstand shear forces in the vascular lumen and short serum half-life, which is essential, since imaging is preformed after the unbound contrast agent has been cleared from the blood [81].

As mentioned above, antibody-based molecular imaging of the brain is restricted to extracerebral markers, since antibodies usually do not spontaneously cross the BBB [72]. However, under brain pathophysiological conditions, such as stroke, multiple sclerosis, or Alzheimer’s disease, the integrity of the BBB is impaired [82], allowing antigen-binding constructs facilitated access to the brain. Several studies have shown that Nbs labelled with fluorochromes or radioligands can access the brain in mouse models of Alzheimer disease [57], glioblastoma [58], and sleeping sickness [83], visualizing intracerebral processes, such as amyloid deposition, tumor-marker (EGFR) expression, and cerebral *Trypanosoma* invasion. Interestingly, Vandesquille et al. could even show that an MRI contrast agent (gadoterate meglumine), which alone does not cross the BBB, is able to pass once bound to a Nb [84]. Further, Li et al. could show that intravenously injected fluorochrome-labelled Nbs can be used to visualize brain amyloid plaques in an Alzheimer’s disease mouse model [57]. However, to date, no Nbs have been utilized to image stroke-induced cerebral inflammation.

## 4. Nanobodies as New Thrombolytic Agents

The only FDA-approved treatment for acute cerebral ischemia is thrombolysis, i.e., the pharmaceutical resolution of occluding blood clots with recombinant tissue plasmin activator (rt-PA). However, in 2009, only 3.4–5.2% of acute stroke patients received this treatment in the USA [85]. Despite recent efforts to extend the therapeutic window with MRI imaging [86], the indications for rt-PA remain limited because of the high risk of bleeding.

Nbs-based thrombolysis may be a promising alternative to rt-PA or might improve its efficacy while simultaneously reducing adverse effects of thrombolysis, such as bleeding [87]. Interestingly, in August 2018, Caplacizumab, a Nb directed against von Willebrand Factor (vWF), was EMA-approved for acquired thrombotic thrombocytopenic purpura (aTTP) [48,88]. Caplacizumab inhibits the interaction of vWF with platelet glycoprotein Ibα (GPIbα) receptors by binding the vWF A1 domain. This reduces platelet adhesion to damaged vessels and thrombus growth without increasing the risk for intracerebral hemorrhage [89]. Momi et al. showed that Caplacizumab is an effective therapy in a guinea pig stroke model. When given up to 15 min after occlusion, Caplacizumab prevented both clot formation and induced reperfusion, thereby reducing brain damage. In contrast to tirofiban (GP-IIb/IIIa-antagonist) and rt-PA, Caplacizumab did not increase intracerebral hemorrhage [90]. Furthermore, vWF inhibition also dampens thrombo-inflammatory processes including leukocyte infiltration [91].

Another potential target for pharmaceutical thrombolysis in stroke is thrombin-activatable fibrinolysis inhibitor (TAFI). TAFI is activated by thrombin or thrombin-thrombomodulin complexes during fibrinolysis. It acts as a negative feedback regulator, i.e., inhibits fibrinolysis. In stroke patients TAFI is elevated in the acute phase of ischemia [92] and is associated with a poor outcome [93]. Furthermore, studies on murine stroke models have shown that anti-TAFI monoclonal antibodies (MA-TCK26D6) reduce fibrinogen deposition, hence improving reperfusion [94]. Nbs against TAFI have been developed. They induce fibrinolysis in vitro and in vivo in a mouse model of thromboembolism [87]. The advantage of Nbs over conventional anti-TAFI antibodies is that Nbs can target different activation states of TAFI [95]. Hence, Nbs not only counteract TAFI activation, but can additionally inhibit already activated TAFI. It remains to be tested if this therapeutic Nb has beneficial effects in stroke.

## 5. Nanobodies to Modulate Post-Stroke Inflammation

The concept of post-stroke inflammation was established a decade ago. However, so far, no studies on Nanobody-based therapy for post-ischemic inflammation have been carried out. Therefore, in this section we will point out possible options to use already existing Nbs as modulators of post-stroke sterile inflammatory processes. The therapeutic approaches discussed include: DAMP inactivation, cytokine neutralization, and inhibition of cell migration.

### 5.1. Targeting DAMP Signaling

Within the first few minutes after stroke onset, DAMPs such as high mobility group protein B1 [9], ATP [10], heat shock protein 70 [11,12], histones, and DNA [13] are released. These molecules play a central role in initiating a sterile innate immune response by binding to corresponding DAMP receptors (including RAGE, P2X7, and Toll-like receptors) [14]. Inhibition of DAMPs and their receptors is a promising therapeutic strategy in stroke. Interestingly, Nbs inhibiting ATP/P2X7 signaling have been generated and successfully tested in two different inflammatory mouse models [96]. During inflammation, ATP is released into the extracellular space by damaged neurons and glial cells. Binding of ATP to P2X7 ion channel induces gating leading to Na^+^/Ca^2+^ influx and K^+^ efflux. This activates the inflammasome, a multiprotein complex that cleaves inactive pro-IL1β into its active form. In stroke patients the release of proinflammatory cytokine IL-1β is associated with poor outcome [97]. Hence, inhibiting P2X7 may be a successful therapeutic approach. However, in vivo preclinical studies show opposing results when it comes to evaluating P2X7 as therapeutic target in stroke. In some studies, P2X7 inhibitors such as Reactive Blue 2 [98], Brilliant Blue G [99], adenosine 5′-triphosphate-2′,3′-dialdehyde (oATP), and A438079 [100] reduced ischemic brain damage in rat stroke models. Conversely, other studies suggest that P2X7 has neuroprotective effects. Kaiser et al. for instance found that P2X7 knockout mice develop worse cerebral edema after experimental stroke [101] and Yanagisawa et al. observed increased brain damage after P2X7 inhibitor (oATP) treatment [102]. Using P2X7 knockout mice and P2X7 inhibitors (oATP, PPADS, and KN62), another group observed that P2X7 had no significant effect on brain damage in experimental stroke [103]. These contradictory results may be attributed to differences in the stroke model, dosage, as well as starting time and duration of P2X7 inhibitor treatment. Furthermore, many of the used inhibitors have a poor specificity for P2X7 [104]. The latter issue may be addressed by using the P2X7-blocking Nbs developed by Danquah et al. [96], since they are highly specific and potent with an IC_50_ in the subnanomolar range. Therefore, they represent valuable tools to further study the role of P2X7 in post-ischemic inflammation.

### 5.2. Inflammatory Cytokine Neutralization

Cytokines are major regulators of post-ischemic sterile inflammation. The main proinflammatory cytokines in stroke are TNFα, IL-1β, and IL-6. In stroke patients, these cytokines rise after occlusion and correlate with neurological outcome [105]. In the following section we will discuss TNFα and IL-1β as two potential targets for Nb-based therapy. In contrast to TNFα and IL-1β, IL-6 mainly has neuroprotective effects [106] and plays a major role in body temperature regulation in stroke patients [107]. Therefore, benefits of interfering with the IL-6 signaling pathway, especially with neutralizing anti-/nanobodies, are of potential negative outcome.

#### 5.2.1. TNFα

In stroke patients, serum TNFα is elevated, peaking at day seven post-ischemia [108] and early TNFα levels in the cerebrospinal fluid (CSF) correlate with neurological outcome [109]. TNFα is mainly produced in macrophages and microglia [110] and binds to TNFα receptors 1 or 2 (TNFR1/2). These receptors initiate several different signaling cascades, e.g., MAPK, NF-κB and caspase 8/10 pathways. Hence, depending on the target cell and the micromilieu, TNFα ligation can lead to inflammation, apoptosis or proliferation [111]. Neutralizing TNFα has different effects depending on the nature (transitory/permanent) and time point of inhibition. Several authors have described that post-ischemic TNFα neutralization significantly reduces the infarct volume in both transient and permanent ischemia models [112,113,114]. On the contrary, Lambertsen et al. showed that TNFα and TNFR knockout mice had larger infarct volumes compared to wild-type mice [115]. Nawashiro et al. demonstrated that low-dose TNFα pretreatment increases the ischemic tolerance [116], suggesting protective effects of TNFα. Targeting TNFα in stroke, therefore, demands careful planning of the time of administration and, ideally, specific inhibitors that only target certain TNFα signaling pathways.

TNFα inhibitors approved by the FDA and EMA are etanercept (TNFR2-Fc-fusion protein), infliximab, adalimumab, golimumab (anti-TNFα monoclonal antibodies), and certizumab (anti-TNFα Fab fragment). Although these inhibitors are currently used to treat autoimmune diseases such as rheumatoid arthritis, they can have severe side effects, such as increased risk for infectious diseases and malignancies, notably lymphomas [117,118]. Another complication during treatment with these biologics is secondary non-response due to the formation of antidrug antibodies [119].

TNFα-targeting Nbs represent a promising alternative that addresses several limitations of the therapeutics listed above. When engineered into dimers or bispecific constructs, TNFα-specific Nbs show a higher potency to neutralize TNFα both in vitro and in mouse models of RA when compared to monoclonal antibodies [118]. Several groups have developed Nbs that inhibit TNFα signaling more selectively, thereby reducing possible adverse effects. Efimor et al. for instance linked antihuman TNFα Nbs to cell lineage marker, such as F4/80 for myeloid cells to neutralize TNFα in a targeted manner [120]. Steeland et al. generated anti-TNFR1 Nbs that selectively inhibit TNFα-TNFR1 interaction, hence sustaining beneficial effects (promoting Tregs) mediated by TNFR2 [121]. This group also tested these Nbs in the EAE mouse model for multiple sclerosis and observed an increase of Nbs in the brain of EAE-induced mice compared to healthy controls [122]. These studies suggest that targeting TNFα signaling with Nbs might be a promising therapeutic approach to dampen post-stroke inflammation.

#### 5.2.2. IL-1β

IL-1β is one of three cytokines in the IL-1 superfamily: IL-1α, IL-1β, and IL-1Ra (IL-1 receptor antagonist). After stroke, the expression of IL-1β, IL-1Ra, and the IL-1 receptors increases [123,124,125]. The two main IL-1 signaling molecules studied in stroke are IL-1β and IL1Ra. IL1Ra competes with IL-1α/β for the IL-1 receptors, thereby inhibiting downstream pro-inflammatory effects [126]. In murine models of stroke, Anakinra, a modified recombinant IL1Ra, improves the neurological outcome, even when administered up to 3 h after onset [127]. Anakinra is FDA-approved for rheumatoid arthritis [128] and phase II trials in stroke patients were successful [129]. In contrast to IL1Ra, IL-1β has detrimental effects in stroke. Preclinical studies with IL-1α/β knockout mice [130] and intrathecal injection of recombinant IL-1β [131] show that IL-1β worsens the neurological outcome. Liberale et al. showed that inhibiting IL-1β with a monoclonal antibody dampens post-ischemic inflammation and reduces infarct size [132]. Canakinumab, a human anti-IL-1β monoclonal antibody, is FDA-approved for arthritis, but remains to be tested in stroke patients. To the best of our knowledge, no Nbs targeting IL-1β or its receptors have been developed so far.

### 5.3. Cell Migration

After stroke onset, leukocytes from the periphery migrate to the ischemic lesion and contribute to post-ischemic inflammation [15]. The most abundant cell type recruited are neutrophil granulocytes, which promote both neuroinflammatory and neuroprotective effects [133]. To this end, regulating cell migration, in particular neutrophil influx, may be a new therapeutic approach to control post-ischemic inflammation.

#### 5.3.1. CXCR2

The CXCR2/CXCL8 axis is involved in chemotaxis of granulocytes and NK cells to the infarcted area after stroke [134]. Targeting this signaling pathway may therefore be beneficial in reducing post-ischemic inflammation. In a rat model of stroke, Connell et al. showed that CXCR1/2 antagonists Repertaxin [135] and G31P [136] significantly reduce ischemic brain damage. Similarly, He et al. suggest that targeting CXCR2 may be beneficial in humans [134]. Interestingly, Brait et al. also found that treatment with a CXCR2 antagonist (SB225002) reduced neutrophil infiltration, but had no effect on neurological outcome [137]. Therefore, the benefits in terms of disease outcome and the optimum time of CXCR2 inhibition to dampen inflammatory effects without limiting regenerative effects of neutrophils still need to be studied. Hereby, Nbs may be a useful instrument, due to their short serum half-life and high specificity. Antihuman CXCR2 Nbs have been developed [138].

#### 5.3.2. CXCR4

Another well-studied chemokine axis in stroke involves CXCR4 and CXCL12. It is associated with both proinflammatory and regenerative processes, including angiogenesis, and the recruitment of neural stem cells and various immune cells to the infarct zone [139]. Inhibiting this pathway with CX549 [140] and AMD3100 [141,142] reduces ischemic brain damage and improves neurological outcome. As for CXCR2, anti-CXCR4 Nbs have been developed [143,144], but have not yet been tested in stroke.

## 6. Conclusions

According to WHO Global Health estimates, strokes are the second leading cause of death worldwide (10.2% of all deaths in 2016). In the future, the prevalence is expected to rise further. This scenario has led to an extensive amount of research in this field. Today, there are many different clinical and preclinical studies evaluating the causes of stroke, diagnostic tools, and possible therapeutic targets. However, despite the extensive research, particularly on post-stroke inflammation, the only treatment for acute ischemic stroke is rt-PA. In part, this can be attributed to the difficult delivery of therapeutics across the BBB. Here, Nbs may be of great benefit. They are small in size, but have a high specificity and can be modified to facilitate crossing of the BBB. Nevertheless, further research has to be undertaken to fully understand which Nb modifications optimize brain penetration and which targets are best suited for Nb-based therapy of stroke.
